# Correction: Antenna Mechanism of Length Control of Actin Cables

**DOI:** 10.1371/journal.pcbi.1004830

**Published:** 2016-03-11

**Authors:** The PLOS Computational Biology Staff

There is a typographical error in Equation 4 in the main text. The equation is also included in Text S1. Please view the correct version of the equation here:

P(l)=(rd)l(koffw)l−1(Γ(koffw+l)Γ(koffw+1))(ekoffrdwkoffr(koff−w)(koffrdw)−(koffw)(Γ(koff−ww)−Γ(−1+koffw,koffrdw))dw2)−1

This equation in reduced form is:

$$P(l)=\frac{{\left(\frac{rk_{off}}{d\;w}\right)}^{\frac{k_{off}}{w}+l-1}\;e^{-(\frac{k_{off}\;r}{d\;w})}\;\Gamma (\frac{k_{off}}{w}-1)}{\Gamma (\frac{k_{off}}{w}+l)}\;{\left(\Gamma (\frac{k_{off}}{w}-1)-\Gamma (\frac{k_{off}}{w}-1,\frac{k_{off}\;r}{d\;w})\right)}^{-1}$$
