# Supplementary material for: Antenna Mechanism of Length Control of Actin Cables
Source: PLoS Comput Biol. 2015 Jun 24;11(6):e1004160. doi: 10.1371/journal.pcbi.1004160 (PMC4480850; doi:10.1371/journal.pcbi.1004160)
Supplement: S1 Text — (DOCX) [file pcbi.1004160.s002.docx]

**Text S1**

**Analytical solution of the master equation**

We used detailed balance to solve the master equation in the regime where the switching rates (parameters $k_{off}$ and$w$) are much larger than rates of assembly and disassembly (parameters $r$ and$d$). Using the detailed balance condition, $P\left( l \right)\bar{r}\left( l \right)=P\left( l+1 \right)d$, where $\bar{r}\left( l \right)$ is the average polymerization rate (see Equation 1), we obtain (for $l>0$ and $w, k_{off},$ $d$ non-zero)

$$P(l) r\frac{k_{off}}{k_{off}+wl}=d P(l+1)$$

By solving this expression recursively we can express $P_{l}$ in terms of$P_{0}$, the probability of zero subunits present at the formin,

$$P(l)= \left( \frac{r}{d} \right)^{l}\prod_{i=0}^{l-1} \left( \frac{k_{off}}{k_{off}+i w} \right) P_{0 .}$$

We use the normalization condition for $P(l)$ to obtain$P_{0}$, which than gives us an simple analytic formula for the length distribution

$$P\left( l \right)= \left( \frac{r}{d} \right)^{l}\frac{\left( \frac{k_{off}}{w} \right)^{l-1}}{\left( \frac{\Gamma\left( \frac{k_{off}}{w}+l \right)}{\Gamma\left( l-1 \right)} \right)} \left( \frac{ⅇ^{\frac{k_{off} r}{d w}}k_{off} r\left( k_{off}-w \right)\left( \frac{k_{off} r}{d w} \right)^{-\left( \frac{k_{off}}{w} \right)}\left( \Gamma\left[ \frac{k_{off}-w}{w} \right]-\Gamma\left[ -1+\frac{k_{off}}{w},\frac{k_{off} r}{d w} \right] \right)}{dw^{2}} \right)^{-1}.$$

where $\Gamma\left( x \right)$is the Gamma function.

We compare this formula to results of simulations in Figure S1. In the fast switching regime where the formula is expected to be valid, we observe agreement with the distribution obtained from simulations. In the regime of slow switching i.e where the switching rates (parameters $k_{off}$ and$w$) are much smaller than rates of assembly and disassembly (parameters $r$ and$d$), the distribution derived from the analytical expression is much narrower than that obtained from simulations, consistent with our intuition that slow switching increases noise.

**Figure S1: Comparison of analytic and numerical distributions.** (A) In the fast switching regime the distribution obtained by detailed balance (blue) matches the cable length distribution obtained from the simulation (red). The parameters of the antenna model used to produce both distributions were$r=0.2, d=0.001, w=10, k_{off}=40$ all in units of s^-1^. (B) When the rates of switching between the on (Smy1 not bound to formin) and off (Smy1 bound to formin) state are slow compared to the rates of polymerization and depolymerisation the analytic and numerical distribution differ. The means of the two distributions are the same while the correct distribution obtained numerically has a larger variance. The parameters used were $r=200, d=100,w=.005, k_{off}=4$ all in units of s^-1^.
